# Supplementary material for: Risk Factors for Same Pathogen Sepsis Readmission Following Hospitalization for Septic Shock
Source: J Clin Med. 2019 Feb 3;8(2):181. doi: 10.3390/jcm8020181 (PMC6406311; doi:10.3390/jcm8020181)
Supplement: Supplementary file 1 [file jcm-08-00181-s001.pdf]

Supplementary Materials

Table S1. Characteristics of Patients with Recurrent Sepsis.

Characteristics	Sepsis due to a Different Organism *n* = 205	Sepsis due to the Same Organism *n* = 69	*p*-Value
Age	65 (58–72)	64 (56–72)	0.658
Male	130 (63.1)	31 (44.9)	0.008
Hypertension	68 (33.0)	31 (44.9)	0.074
Stroke	22 (10.7)	2 (2.9)	0.047
Diabetes	57 (27.7)	19 (27.5)	0.983
Coronary artery disease	30 (14.6)	5 (7.2)	0.114
Chronic pulmonary disease	50 (24.3)	13 (18.8)	0.353
Chronic renal failure	12 (5.8)	7 (10.1)	0.221
Liver cirrhosis	33 (16.0)	18 (26.1)	0.063
Malignancy	165 (80.1)	58 (84.1)	0.467
28 d mortality	5 (2.4)	1 (1.4)	0.630
90 d mortality	11 (5.3)	5 (7.2)	0.558
Mechanical ventilation	12 (5.8)	2 (2.9)	0.338
ICU stay	0 (0–2)	0 (0–4)	0.763
Same site	135 (65.5)	62 (89.9)	<0.001

Table S2. Characteristics of Causal Organisms.

Characteristics	Total (%) *n* = 274	Different Organism (%) *n* = 205	Same Organism (%) *n* = 69	*p*-Value
**Organism**				
Gram-positive	34 (12.4)	32 (15.5)	2 (2.9)	0.006
Gram-negative	181 (65.8)	115 (55.8)	66 (95.7)	<0.001
Viral	13 (4.7)	13 (6.3)	0 (0)	0.033
Fungi	4 (1.5)	3 (1.5)	1 (1.4)	0.997
**Site**				
Urinary	47 (17.1)	23 (11.2)	24 (34.8)	<0.001
Gastrointestinal	146 (53.1)	105 (51.0)	41 (59.4)	0.224
Respiratory	73 (26.5)	70 (34.0)	3 (4.3)	<0.001
Skin and soft tissue	10 (3.6)	8 (3.9)	2 (2.9)	0.705
Other	29 (10.5)	23 (11.2)	6 (8.7)	0.563
